# Downregulated Expression of Virulence Factors Induced by Benzyl Isothiocyanate in *Staphylococcus Aureus*: A Transcriptomic Analysis

**DOI:** 10.3390/ijms20215441

**Published:** 2019-10-31

**Authors:** Xiaoning Wang, Hongyan Wu, Tongxin Niu, Jingran Bi, Hongman Hou, Hongshun Hao, Gongliang Zhang

**Affiliations:** 1 School of Food Science and Technology, Dalian Polytechnic University, Dalian 116034, Chinahouhongman2011@hotmail.com (H.H.); 2 Graduate School of Environmental and Life Science, Okayama University, Okayama 700-8530, Japan; wuhongyan1908@hotmail.com; 3 Department of Inorganic Nonmetallic Materials Engineering, Dalian Polytechnic University, Dalian 116034, China,

**Keywords:** *Staphylococcus aureus*, benzyl isothiocyanate, RNA-seq, differentially expressed genes, biofilm

## Abstract

*Staphylococcus aureus* (*S. aureus*) is a common foodborne pathogen that leads to various diseases; therefore, we urgently need to identify different means to control this harmful pathogen in food. In this study, we monitored the transcriptional changes of *S. aureus* by RNA-seq analysis to better understand the effect of benzyl isothiocyanate (BITC) on the virulence inhibition of *S. aureus* and determined the bacteriostatic effect of BITC at subinhibitory concentrations. Our results revealed that, compared with the control group (SAC), the BITC-treated experimental group (SAQ_BITC) had 708 differentially expressed genes (DEGs), of which 333 genes were downregulated and the capsular polysaccharide (*cp*) was significantly downregulated. Furthermore, we screened five of the most virulent factors of *S. aureus*, including the capsular polysaccharide biosynthesis protein (*cp5D*), capsular polysaccharide synthesis enzyme (*cp8F*), thermonuclease (*nuc*), clumping factor (*clf*), and protein A (*spa*), and verified the accuracy of these significantly downregulated genes by qRT-PCR. At the same time, we used light microscopy, scanning electron microscopy (SEM) and inverted fluorescence microscopy (IFM) to observe changes in biofilm associated with the *cp5D* and *cp8F*. Therefore, these results will help to further study the basis of BITC for the antibacterial action of foodborne pathogenic bacteria.

## 1. Introduction

*Staphylococcus aureus* (*S. aureus*) is a gram-positive bacterium of the symbiotic flora of humans and various animal species and is a common foodborne pathogen [1,2]. Today, foodborne illness has become one of the major food safety and public health issues caused by pathogenic microorganisms in food and is one of the leading causes of morbidity and mortality worldwide [3]. The high density of foodborne diseases and even food poisoning caused by *S. aureus* infection threatens human health and safety. Therefore, controlling microbes in food, especially *S. aureus*, remains a worldwide problem [4]. *S. aureus* has a wide range of viability, and this strong viability contributes to disease [5,6]. That is, the inhibition of *S. aureus* provides an important basis for foodborne microbial control.

So far, people have been increasingly studying bacteriostatic agents, and the research on *S. aureus* has not stopped. For example, Yunbin Zhang et al. found that cinnamon essential oil exhibited effective antibacterial activity against *Escherichia coli* and *S. aureus* by scanning electron microscopy to observe cell microstructure, cell membrane permeability and integrity [7]. For another example, Jiamu Kang et al. studied the antibacterial mechanism of peppermint essential oil (PEO) and the activity of *S. aureus* biofilm and found that PEO can significantly inhibit the formation of biofilm [8]. Among cruciferous vegetables, isothiocyanate (ITC) is a relatively common organic sulfur compound that has been extensively studied and naturally exists as a glucosinolate [9,10]. ITC has high anticancer properties and inhibits cell proliferation [11,12]. The mechanism by which ITC inhibits cancer cell proliferation is usually achieved by inhibiting proteins in the process of tumor initiation and proliferation [12]. In recent years, researchers have begun to study the effects of benzyl isothiocyanate (BITC) on bacteria. For example, Dufour studied the antibacterial effect of BITC on *Vibrio parahaemolyticus* [13], and Jie Song studied the inhibition and bacterial mechanism of BITC on *Vibrio parahaemolyticus* at the transcriptional level [14]. However, the effect of BITC on *S. aureus* at the transcriptional level has not yet been analyzed.

There are an increasing number of studies on the transcriptome analysis of *S. aureus*, such as the analysis of the formation of *S. aureus* biofilm in the presence of sublethal concentrations of disinfectants and the validation of related genes, including cell factors (*clfAB*) and capsular polysaccharides (*cap8EFGL*) [15]. In another example, resveratrol acted as a natural phytoalexin against *S. aureus* at subinhibitory concentrations and was subjected to transcriptome analysis. The results showed that resveratrol also reduced the expression of α-hemolysin under the premise of inhibiting the normal growth of *S. aureus* [16]. The virulence component of *S. aureus* is the main cause of pathogenicity, including extracellular capsular polysaccharides (CPs), related adhesins, exoenzymes, and exotoxins [17,18]. For example, thermonuclease (*nuc*) is a relatively common virulence gene, and *S. aureus* nucleases are considered to be important virulence factors and unique markers widely used to detect *S. aureus* from food samples and clinical specimens [19,20,21]. Capsular polysaccharide (CP) is a major virulence factor that strengthens resistance against phagocytic uptake by human polymorphonuclear leukocytes [21,22].

A biofilm is a structured community of bacterial cells that are enclosed in a self-produced polymer matrix that adheres to glass or other surfaces by a matrix, and protected in a growth mode that can survive in harsh environments [23]. According to Salimena et al., CP production and biofilm formation of *S. aureus* isolated from milk from three different Brazilian regions were studied, and CP and biofilm formation were obtained in vitro, and the capsular genotype and phenotype were found. There is a significant correlation with the amount of biofilm formation, and *cap5* isolates tend to form more biofilms than *cap8* isolates [24].

Therefore, to study the inhibition mechanism of BITC against *S. aureus* at a deeper level, we analyzed the transcriptome level and further verified the expression of different virulence genes by qRT-PCR to understand the virulence mechanism of BITC on *S. aureus*. In addition, the qualitative analysis of the effect of BITC on *S. aureus* biofilm further verified the downregulated expression of *CP* gene and the effectiveness of BITC.

## 2. Results

### 2.1. Antibacterial Assay 

The minimum inhibitory concentration (MIC) of BITC against *S. aureus* was 0.5 mmol/L.

### 2.2. Transcriptome Library Construction and Sequencing Data Quality Control 

To obtain a comprehensive analysis of the effect of BITC on *S. aureus*, six cDNA libraries were constructed after treating *S. aureus* with BITC, and each library was sequenced with Illumina HiSeqTM 2500, which is with a minimum depth of 2× reads in sequencing. We generated a total of 108,923,888 raw reads and 106,407,410 clean reads. The average error rate for all data was less than 0.03%, while Q20 > 97% and Q30 > 92% in each pool indicated that the data quality assessment was acceptable (Table S1).

### 2.3. Reads and Reference Genome Comparison 

We found that 95.78–97.15% of the reads were perfectly matched to the reference genome in each library. For the unique mapped reads and the multi-mapped reads, 89.59–92.62% and 4.52–5.83% (16,510,649–21,860,562) were matched, respectively (Table 1). 

### 2.4. Analysis of Differentially Expressed Genes (DEGs) 

**Figure 1:**
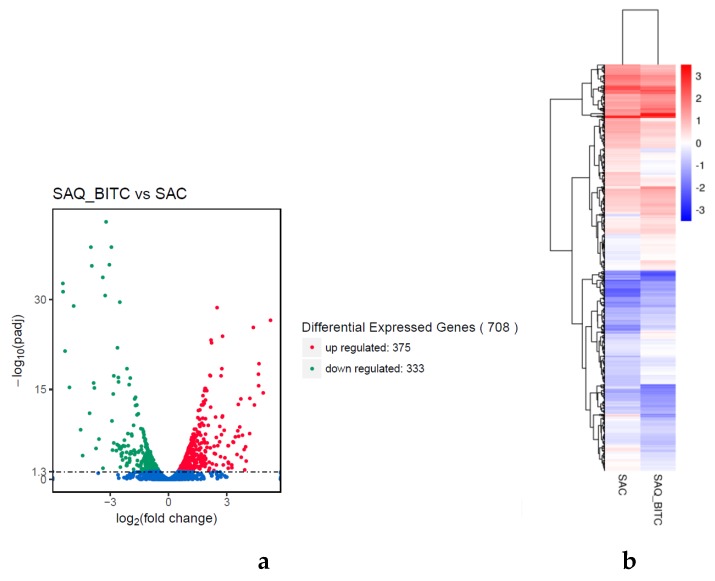
Volcano map of differentially expressed genes (DEGs) (**a**) and hierarchical clustering analysis of DEGs for SAQ_BITC vs SAC (**b**). Red, green, and blue indicate upregulated, downregulated, and no significant changes in DEGs, respectively. Among them, two columns (from left to right) represent SAC and SAQ_BITC, respectively, different rows represent different genes, and different colors represent different gene expression levels of the two groups of samples.

The *S. aureus* cells treated with BITC at 1/4 MIC were cultured for 9 h. The experimental group (SAQ_BITC) was compared with the BITC-free control group (SAC) to evaluate the transcription of *S. aureus* under BITC treatment. The selection criteria were p < 0.05. We identified a total of 708 significant differentially expressed genes (DEGs) in SAQ_BITC. Among them, 333 genes were downregulated, and 375 genes were upregulated (Figure 1a).

To obtain expression patterns of the DEGs under the SAQ_BITC and SAC conditions, genes having similar metabolic functions or involved in similar cellular pathways under different conditions were used to speculate the functions of unknown genes with novel characteristics. In order to easily decide on the number of clusters from the dendrogram and more intuitively indicate the results of cluster analysis of DEG, we used the agglomerative hierarchical clustering analysis (Figure 1b). The FPKM values of the DEGs under the two experimental conditions were used as the expression level. The regions of different colors represent different clusters. Class grouping information, which is similar to gene expression patterns in the same group, can have similar or identical biological processes.

**Figure 2:**
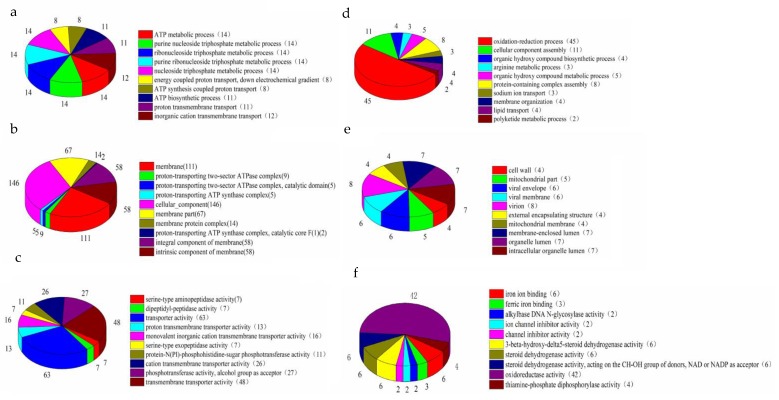
Analysis of the most significant gene functions of SAQ_BITC compared to SAC. We analyzed the functional effects of significant DEGs that were upregulated including (**a**) biological processes (BP), (**b**) cell components (CC), and (**c**) molecular functions (MF); and downregulated, including (**d**) biological processes (BP), (**e**) cell components (CC), and (**f**) molecular functions (MF).

We classified DEGs by Gene Ontology (GO) enrichment analysis, which included three categories related to virulence: “Biological processes (BP)”, “cell components (CC)” and “molecular functions (MF)”. To visually reflect the GO category, we compared SAQ_BITC with SAC, selected the most significant 30 GO term enrichment results, and found that 537 DEGs were annotated to 23 biological processes, two cellular components and five molecular functions (Figure 2).

**Figure 3:**
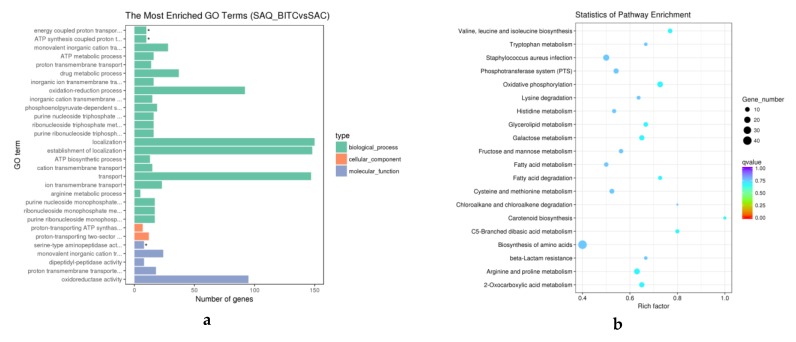
Functional enrichment of DEGs with GO categorization (**a**) and the KEGG differentially expressed gene enrichment analysis scatter plot (**b**). The figure shows the most significant 30 GO term enriched genes and 20 significant KEGG pathways were selected for enrichment, and the Rich factor was used to identify the extent of enrichment.

We selected the top 10 GO functions with the most significant enrichment in the three GO categories (Figure 3a). Among the downregulated GO functions, the oxidation-reduction process was the most significantly enriched biological process and had the largest proportion of DEGs. The cell wall was the most prominent in the cell component class, and iron ion-binding was most prominent in molecular function. At the same time, on the basis of upregulation, the ATP metabolic process was highly enriched among the biological processes and had 14 differential gene enrichments. The membrane fraction was significantly enriched in the cell fraction and had 111 differential genes. Serine-type aminopeptidase activity was significantly enriched in molecular function.

To further investigate the metabolic pathways of *S. aureus* treated with BITC, KEGG enrichment analysis of differential genes was performed. We selected the 20 most significantly enriched path entries as shown in Figure 3b. The selection criteria were p-value and the value of q-value; the closer the values were to zero, the more significant the enrichment. Among them, oxidative phosphorylation, arginine and proline metabolism, valine, leucine and isoleucine biosynthesis, and the 2-oxocarboxylic acid metabolism pathway were the most significantly enriched.

### 2.5. Validation of Virulence-Related Gene Results by qRT-PCR

**Figure 4:**
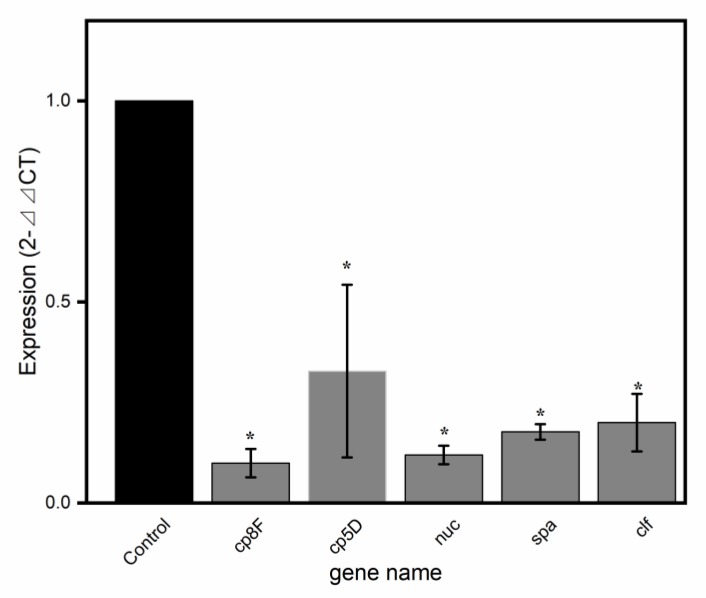
The expression of the differentially expressed genes (DEGs) verified by qRT-PCR. Relative expression of *cp8F*, *cp5D*, *nuc*, *spa* and *clf* compared to the 16S rRNA normalized to one control. The resulting data were derived from the average of three independent replicates. * showed significant differences in gene expression (*p* < 0.01).

qRT-PCR is commonly used as a method for verifying the accuracy of differential gene expression in RNA-seq analysis. Five common and representative virulence genes were screened for qRT-PCR validation in SAQ_BITC. The DEG validation results were identical to those obtained by RNA-Seq analysis (Table 2; Figure 4). Compared with RNA-Seq, the scale of expression of the five genes in the qRT-PCR analysis was similar, indicating that the DEGs were successfully identified by RNA-Seq.

### 2.6. Effect of BITC on the S. Aureus Biofilm

**Figure 5:**
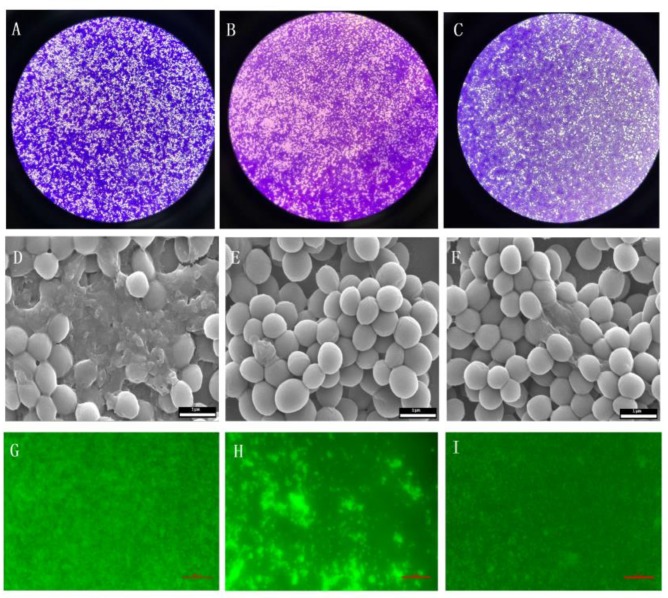
The effect of BITC on the biofilm formation of *S. aureu*s was observed by light microscopy (40×) (**A**, control; **B**, 1/4 MIC-BITC; **C**, 1/8 MIC-BITC), SEM image of *S. aureus* biofilm (20,000×) (**D**, control; **E**, 1/4 MIC-BITC; **F**, 1/8 MIC-BITC), IFM image of *S. aureus* biofilm (excitation wavelength 488 nm, blocking laser chip wavelength 515 nm) (**G**, control; **H**, 1/4 MIC-BITC; **I**, 1/8 MIC-BITC).

Firstly, the existence of biofilms was determined by the light microscopy. Then the effect of BITC on the inhibition of *S. aureus* biofilm was further observed by SEM and IFM. The results showed that, as shown in the Figure 5, a thick biofilm coating could be detected without treatment with BITC compared to the control group. However, the images treated with 1/4 MIC BITC and 1/8 MIC showed a marked decrease in microbial adhesion and a marked decrease in microbial adhesion after high concentration treatment. As shown, the effect of BITC on *S. aureus* biofilm was further confirmed by IFM.

## 3. Discussion

Foodborne pathogens are a very dangerous biological threat because billions of gastrointestinal diseases are caused by foodborne pathogens worldwide, leading to more than five million deaths [16,25]. *S. aureus*, a common foodborne pathogen, is one of the main pathogens of food poisoning, can be found in a variety of foods, and can cause varying degrees of gastroenteritis when food is contaminated [26,27]. Previous studies have shown that *S. aureus* cells are subject to certain damage under low temperature conditions, but do not die [15,27]. Therefore, *S. aureus* poses a very serious threat to food safety [28]. BITC, a kind of edible flavor, was discovered to have a certain antibacterial effect. For example, Jie Song et al. found that BITC had strong antibacterial activity against *Vibrio parahaemolyticus* [14].

Transcriptomic analysis is gradually being widely used because it can comprehensively evaluate differential genes and enrichment pathways in samples. In recent years, many studies have shown that transcriptome analysis and qRT-PCR can comprehensively analyze *S. aureus*. For example, Slany M. et al. analyzed the formation of *S. aureus* biofilm at the transcriptome level by adding a sublethal dose of disinfectant [15]. Another example is the virulence analysis of *S. aureus* strains isolated from animals by Zahid Iqbal et al. [29].

Similar to other bacterial pathogens, *S. aureus* expresses capsular polysaccharide (*cp*) with two major types of capsular polysaccharides, namely, CP5 and CP8, which are present in all clinical *S. aureus* strains. Capsular polysaccharides are a major virulence factor that enable *S. aureus* to avoid swallowing and killing [22,30,31]. Biofilm formation has varying degrees of association with the *cp5D* and *cp8F*. And BITC has a significant inhibitory effect on the biofilm production of *S. aureus* effectively observed by SEM and IFM. This is consistent with the expression of the *cp5D* and *cp8F* genes. In addition, the aggregation factor (clf) in the adhesion gene is also closely related to biofilm formation, and its protein mediates adhesion to fibrinogen [32,33]. As a typical virulence factor, protein A may have a major impact on osteoclast differentiation in the early stages of *S. aureus* infection and is now considered to be useful as a preventative for bone damage during *S. aureus* osteomyelitis [34,35]. Thermonuclease (*nuc*) is a special *S. aureus* virulence factor and is widely used in sample testing [20,21]. Recent studies have found new nucleases that are complementary to nuc from *S. aureus* [36]. Therefore, in future we need to pay more attention to these regulatory genes [37]. BITC effectively reduces the expression of these virulence factors.

In this study, the analysis of the effect of BITC on *S. aureus* revealed potential control mechanisms and the possible application value of BITC. Further research on protein levels and bacterial morphology is needed to validate specific functions and potential interactions at the molecular level.

## 4. Materials and Methods 

### 4.1. Bacterial Strains and Culture

*Staphylococcus aureus* ATCC 6538 selected in the study was obtained from the Food Microbiology Laboratory of the Dalian Polytechnic University (Dalian, China) and kept at −80 °C. Prior to use, the bacteria were activated twice in lysogenic fermentation broth (LB) medium at 37 °C for 12 h or more. 

### 4.2. Antibacterial Assays

Determination of the MIC was performed with the broth microdilution method [38]. Different dilutions of BITC and bacterial cultures were added to sterile 96-well microtiter plates to culture at 37 °C for 12 h. Mueller-Hinton Broth (MHB) with or without bacterial cultures served as the control.

### 4.3. Extraction and Detection of RNA Samples

In this study, the BITC-treated bacterial solution was cultured to a stable growth phase, followed by RNA extraction. The total RNA of the sample was then extracted using an RNAprep Pure Cell/Bacterial Kit (Tiangen Biotech, Beijing, China). The degree of RNA degradation and contamination was analyzed by agarose gel electrophoresis. At the same time, the ratio of OD260/280 was used to verify the purity of the six RNA samples. Qubit accurately quantified the RNA concentration, and the RNA integrity was accurately detected with an Agilent 2100 (G2939B, Agilent Technologies, Palo Alto, CA, USA). Finally, the extracted RNA samples were stored at −80 °C until use. 

### 4.4. Library Construction and Sequencing 

Six RNA samples were used as the initial input material for the library. A new sequencing library was formed using the specific NEBNext UltraTM Directional RNA Library Preparation Kit (NEB, Ipswich, MA, USA) according to the manufacturer’s recommendations, and an index code was then added to the attribute sequence of each sample. That is, the rRNA was removed using a special kit. The purified cDNA fragments were then purified by the A-tail and ligated sequencing linker, which were added by the end-end repair, using the AMPure XP system (Beckman Coulter, Beverly, MA, USA). Three microliters of USER enzyme (NEB, USA) was then reacted with the cDNA and subjected to PCR. The reaction was then carried out using Index (X) Primer, Phusion High-Fidelity DNA polymerase and universal PCR primers. Finally, the Agilent Bioanalyzer 2100 system (G2939B, Agilent Technologies, Palo Alto, CA, USA) was used to evaluate the quality of the products and libraries.

### 4.5. Biological Information Analysis

After obtaining the original sequencing sequence by building a library and high-throughput sequencing (Illumina HiSeq^TM^ 2500 (Illumina, CA, USA), the sequencing data were evaluated for quality. The filtered sequencing sequences were then subjected to genomic localization analysis and reference sequence alignment analysis. In the case of a related species reference sequence or reference genome, bioinformatics analysis was performed by including reads containing adapters, low quality reads and yield-N readings (clean reads), and removing clean data from the raw data. At the same time, the GC content, Q20 (percentage of bases with a Phred value > 20) and Q30 (percentage of bases with a Phred value > 30) were obtained from the data, and all downstream analyses were performed with the high-quality clean data. The clean reads were aligned with reference sequences to obtain an alignment rate using bowtie2 with default parameters. To assess gene expression levels, we chose the common method FPKM (expected number of fragments per kilobase of transcript sequence per million base pairs sequenced). A log2 (fold change) <1 was used to select the downregulated DEGs. Then, GO enrichment analysis was performed with software for the GO enrichment analysis and p < 0.05 indicated the DEGs that were significantly enriched. The KEGG enrichment classification, the enrichment classification of biological functionals and metabolic pathways, identified the major pathways the DEGs were involved in through determining the significantly enriched metabolic pathways.

### 4.6. qRT-PCR Validation of Differential Genes

To ensure the accuracy of the RNA-seq data results, we extracted RNA from the SAC control and SAQ_BITC experimental samples, each set in triplicate. According to the instructions of the PrimeScriptTM RT Kit with gDNA Eraser (TaKaRa, Otsu, Japan), modifications were performed to remove impurities and for reverse transcription into cDNA templates, and the resulting samples were placed at −20 °C for later use. The 16S rRNA gene was used as an endogenous gene, and the specific primers for the differential genes screened by RNA-Seq were designed using Primer 5.0 software and are listed in Table 3. Amplification was performed according to the TransStart Top Green qPCR SuperMix Kit (TransGen Biotech, BeiJing, China) in a 20 μL system. Finally, the differential gene expression level was evaluated using the 2^−ΔΔCt^ method [39]. Significant analysis was performed using Student’s *t*-test. A significance level of *p* < 0.05 was considered to be significant.

### 4.7. Effects of BITC on Formation of S. Aureus Biofilm

For *S. aureus* biofilm, place coverslips in a 6-well microtiter plate and add overnight cultured *S. aureus* and certain nutrients, add different concentrations of BITC to final concentrations of 1/4 MIC and 1/8 MIC to culture for 24 h. Add MHB as a blank control. The coverslips were washed three times with PBS, then dried at room temperature, and the biofilm was stained with 0.1% crystal violet or 0.01% acridine orange for 15 minutes. Thereafter, the biofilm was observed under a light microscope (Nikon, Tokyo Japan) or IFM (Nikon, Tokyo, Japan), respectively. For SEM, the cultured biofilm was washed three times with PBS, and then the biofilm on the coverslip was fixed with 2.5% glutaraldehyde and dehydrated with ethanol (50%, 70%, 80%, 90% and 100%). The dried slides were glued to the table and sprayed with gold and then observed under SEM (Quanta 450, Waltham, MA, USA) [40].

## 5. Conclusions

Analysis of the antibacterial mechanism of foodborne pathogens by adding flavorants is a very important parameter in food safety. For the first time, the study screened virulence-related differential genes by RNA-seq and further analyzed the expression of differential genes. Through the analysis of differential genes at the molecular level, further changes in protein and morphological levels are needed to verify the virulence changes in *S. aureus*. These results provide a certain data basis for the antibacterial aspect of *S. aureus*.

## Figures and Tables

**Table 1:** Summary of RNA-seq alignment.

Sample Name	Total Reads	Total Mapped	Multiple Mapped	Uniquely Mapped
SAC1	17,425,772	16,699,263 (95.83%)	912,234 (5.23%)	15,787,029 (90.6%)
SAC2	19,641,306	18,949,431 (96.48%)	995,830 (5.07%)	17,953,601 (91.41%)
SAC3	19,752,162	18,917,775 (95.78%)	1,151,497 (5.83%)	17,766,278 (89.95%)
SAQ_BITC1	17,015,102	16,523,451 (97.11%)	782,977 (4.6%)	15,740,474 (92.51%)
SAQ_BITC2	19,245,460	18,643,857 (96.87%)	928,383 (4.82%)	17,715,474 (92.05%)
SAQ_BITC3	13,327,608	12,947,341 (97.15%)	602,910 (4.52%)	12,344,431 (92.62%)

**Table 2:** Data of the differentially expressed genes.

Gene_Id	Gene Name	log2FoldChange (SAQ_BITC vs SAC)	Pval (SAQ_BITC vs SAC)	Padj (SAQ_BITC vs SAC)	Significant (SAQ_BITC vs SAC)
SAOUHSC_00119	*Cp8F*	−3.9945	2.33 × 10^−42^	2.02 × 10^−39^	DOWN
SAOUHSC_00117	*CP5D*	−5.4282	1.73 × 10^−34^	5.63 × 10^−32^	DOWN
SAOUHSC_00818	*nuc*	−2.1358	2.85 × 10^−21^	3.53 × 10^−19^	DOWN
SAOUHSC_00069	*spa*	−1.55	2.54 × 10^−13^	1.10 × 10^−11^	DOWN
SAOUHSC_00812	*clf*	−1.1057	2.92 × 10^−7^	4.77 × 10^−6^	DOWN

**Table 3:** Primer sequences for qRT-PCR.

Gene	Primer	Sequence (5’–3’)
*16S rRNA*	16S rRNA-F	CGTGCTACAATGGACAATACA
	16S rRNA-R	ACAATCCGAACTGAGAACAAC
*Cp8F*	Cp8F-F	ACAGACTTTAGTTATCCCTTAC
	Cp8F-R	TGATGCCAGTGATTACCTTTA
*CP5D*	CP5D-F	CTTTAGTTGTTGGTGCTGGTC
	CP5D-R	CGGTTCAAGTTTCATTTCGTC
*nuc*	nuc-F	GAAAGGGCAATACGCAAAG
	nuc-R	ACGCCATTATCTGTTTG
*spa*	spa-F	ATAAGAAGCAACCAGCAAAC
	spa-R	GGCTAATGATAATCCACCAA
*clf*	clf-F	ACGAATGGCGATGTTGTAGC
	clf-R	CTCGGTCTGTAAATAAAGGTAATG

## Supplementary Materials

Supplementary materials can be found at https://www.mdpi.com/1422-0067/20/21/5441/s1.

## Abbreviations

ITCs	isothiocyanates
BITC	benzyl isothiocyanate
BP	biological process
CC	cellular component
MF	molecular function
DEGs	differential expression genes
FPKMs	Fragments Per Kilobase of exon model per Million mapped reads
GO	Gene Ontology
KEGG	Kyoto Encyclopedia of Genes and Genomes
qRT-PCR	quantitative Real-time Polymerase Chain Reaction
