# Supplementary material for: Downregulated Expression of Virulence Factors Induced by Benzyl Isothiocyanate in Staphylococcus Aureus: A Transcriptomic Analysis
Source: Int J Mol Sci. 2019 Oct 31;20(21):5441. doi: 10.3390/ijms20215441 (PMC6862589; doi:10.3390/ijms20215441)
Supplement: Supplementary file 1 [file ijms-20-05441-s001.pdf]

**Table S1** Quality assessment of sample sequencing data

| Sample name | Raw Reads | Clean Reads | Clean Bases(Gb) | Error(%) | Q20(%) | Q30(%) | GC(%) |
|---|---|---|---|---|---|---|---|
| SAC1 | 17719104 | 17425772 | 2.61G | 0.03 | 97.45 | 92.72 | 35.3 |
| SAC2 | 20159534 | 19641306 | 2.95G | 0.03 | 97.33 | 92.45 | 35.85 |
| SAC3 | 20159288 | 19752162 | 2.96G | 0.03 | 97.47 | 92.77 | 35.21 |
| SAQ_BITC1 | 17509930 | 17015102 | 2.55G | 0.03 | 97.17 | 92.09 | 35.14 |
| SAQ_BITC2 | 19672798 | 19245460 | 2.89G | 0.03 | 97.24 | 92.26 | 35.37 |
| SAQ_BITC3 | 13703234 | 13327608 | 2G | 0.03 | 97.45 | 92.71 | 35.43 |
